# P-676. Risk Factors Associated with Longer Hospital Stay in Elderly Patients with Respiratory Syncytial Virus Infections

**DOI:** 10.1093/ofid/ofae631.872

**Published:** 2025-01-29

**Authors:** Ashish Bhargava, Susan M Szpunar, Mamta Sharma, Louis Saravolatz

**Affiliations:** Ascension St. John Hospital; Wayne State University School of Medicine, Grosse Pointe Woods, Michigan; Ascension St. John Hospital, MI; Henry Ford St John Hospital, Grosse Pointe Woods, MI. 48236, Michigan; Ascension St John Hospital, gross pointe woods, Michigan

## Abstract

**Background:**

Annually, 3-7% of healthy older patients and 4-10% of high-risk adults develop respiratory syncytial virus (RSV) infections. Elderly patients above 65 years of age, with congestive heart failure, chronic lung disease, or weakened immune systems are at high risk for severe RSV infections. Little is known about the factors that are associated with the length of hospital stay (LOS) among these elderly individuals.

**Methods:**

A multicenter historical cohort study was conducted on elderly patients ( >65 years of age) hospitalized for laboratory-confirmed RSV-related diseases in Ascension hospitals in Southeast Michigan between January 2017 and December 2022. Hospitalized patients were identified using ICD 10 codes for RSV-related diseases. Electronic medical records were reviewed after IRB approval. LOS was categorized as below the mean LOS or at the mean and above. Data were analyzed using Student’s t-test, the chi-squared test, the Mann-Whitney U test and logistic regression using SPSS v. 29.0.

**Results:**

Of 239 patients, the mean (sd) age of the cohort was 78.3 + 8.4 years, 157 (65.7%) were female and 176 (73.6%) were white. The mean body mass index (BMI) was 29.5 + 8.8 kg/m^2^. The mean Charlson Weighted Index of Comorbidity (CWIC) score was 2.4 + 2.0. The mean hospital LOS was 7.5 + 5.1 days. Prolonged LOS was noted in 89 (37.2%) patients. Factors associated with longer LOS ( >7.5) in univariable analysis were chronic lung disease, solid tumors, CWIC score, smoking status, home oxygen, oxygen requirement during hospitalization, qSOFA, abnormal admission chest x-ray (CXR), lower respiratory tract infection, and respiratory failure requiring intubation. The multivariable logistic regression revealed that predictors for prolonged LOS among elderly patients were smoking status (OR, 2.2; 95% CI 1.2-4.0), abnormal admission CXR (OR, 2.1; 95% CI 1.2-3.7), and respiratory failure requiring intubation (OR, 9.4; 95% CI 2.6-34.4).

**Conclusion:**

Our study finds that patient’s smoking status, abnormal admission CXR and respiratory failure requiring intubation were significantly associated with prolonged LOS among elderly patients with RSV infections. Knowing these risk factors may help identify patients who would benefit from early interventions to mitigate the duration of hospitalization.

**Disclosures:**

**All Authors**: No reported disclosures

